# Supercritical Solution Impregnation: A Strategy to Prevent Prosthetic Infections

**DOI:** 10.1002/gch2.70148

**Published:** 2026-09-12

**Authors:** Andrea Salas, Sara Díaz, Eduardo Pérez, Helga K. Ruiz, Rama Hafian, Pablo Sanz, Lourdes Calvo, Albertina Cabañas

**Affiliations:** ^1^ Department of Physical Chemistry Complutense University of Madrid Madrid Spain; ^2^ Department of Chemical Engineering and Materials Complutense University of Madrid Madrid Spain; ^3^ Hospital General Universitario Gregorio Marañón Marañón Spain

**Keywords:** antibacterial biomaterials, biofilm prevention, drug impregnation, orthopaedic prosthesis, supercritical carbon dioxide

## Abstract

One of the most devastating and frustrating complications in orthopaedic surgery is infection. A project is underway in our laboratory to explore the use of supercritical CO_2_ (scCO_2_) as an impregnation medium for treating orthopaedic prostheses to prevent infections. We present our results on the incorporation of antibiotics into the polymeric part of prostheses (inserts) using scCO_2_ as a green solvent capable of penetrating the inserts carrying the drug without altering their structural integrity. Experiments were performed using pieces of actual knee joint liners of cross‐linked ultra‐high molecular weight polyethylene (UHMWPE) cut into small pieces. Materials were impregnated with the antibiotic clindamycin dissolved in CO_2_ at 35–55°C and 100–200 bar. Moderate loadings (0.13%–1.85% mass) were obtained due to the compact nature of the UHMWPE liners and the weak interaction between the drug and the support. Drug loading increased with the CO_2_ density and the depressurization rate. The impregnated prosthesis pieces were free of solvent residues and exhibited antibacterial activity against *S. epidermidis*, evidenced by the formation of inhibition halos, confirming the effectiveness of this approach in preventing bacterial colonization.

## Graphical abstract

Infections following hip or knee replacement surgery are painful, difficult to treat, and often require additional operations. This study presents a sustainable carbon dioxide‐based method for loading antibiotics into prosthetic materials. These medicated prostheses prevent bacterial growth without altering implant properties, potentially reducing infections, revision surgeries, healthcare costs, and recovery time.

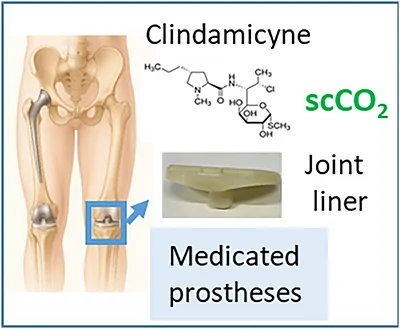

## 1 Introduction

As global life expectancy continues to rise, the frequency of orthopaedic surgeries is also increasing. Among these, hip and knee replacements are one of the most common procedures, primarily indicated for the treatment of osteoarthritis [1]. According to the Organisation for Economic Co‐operation and Development (OECD), in 2023 the number of hip and knee replacements per 100,000 inhabitants was 172 and 119, respectively [2]. Furthermore, a recent projection model for primary hip and knee arthroplasty forecasts a dramatic increase over the next few decades, exceeding 500%, due to ageing populations and rising osteoarthritis rates [3].

On average, the cost of a hip or knee replacement surgery in the US ranges from $30,000 to $50,000 [4], depending on several factors, including location and complexity of the surgery, so prosthesis surgeries present a major cost burden.

After orthopaedic surgery, the most devastating complication is infection. Most bacterial infections in prostheses are caused by local microorganisms that can be resistant to the host's immune system, allowing bacteria to win the “race for the implant surface” in the early moments and create biofilms [5]. Thus, when this biofilm is mature, the only effective treatment to remove it is an additional surgery to replace the prosthesis, combined with systemic and local antibiotic treatment [6]. This causes severe morbidity to patients and has a high economic impact [7]. The use of drug‐eluting implants as a method to prevent infections has been previously proposed. Medicated prostheses offer potential to reduce complications and revision surgeries [8, 9].

**FIGURE 1:**
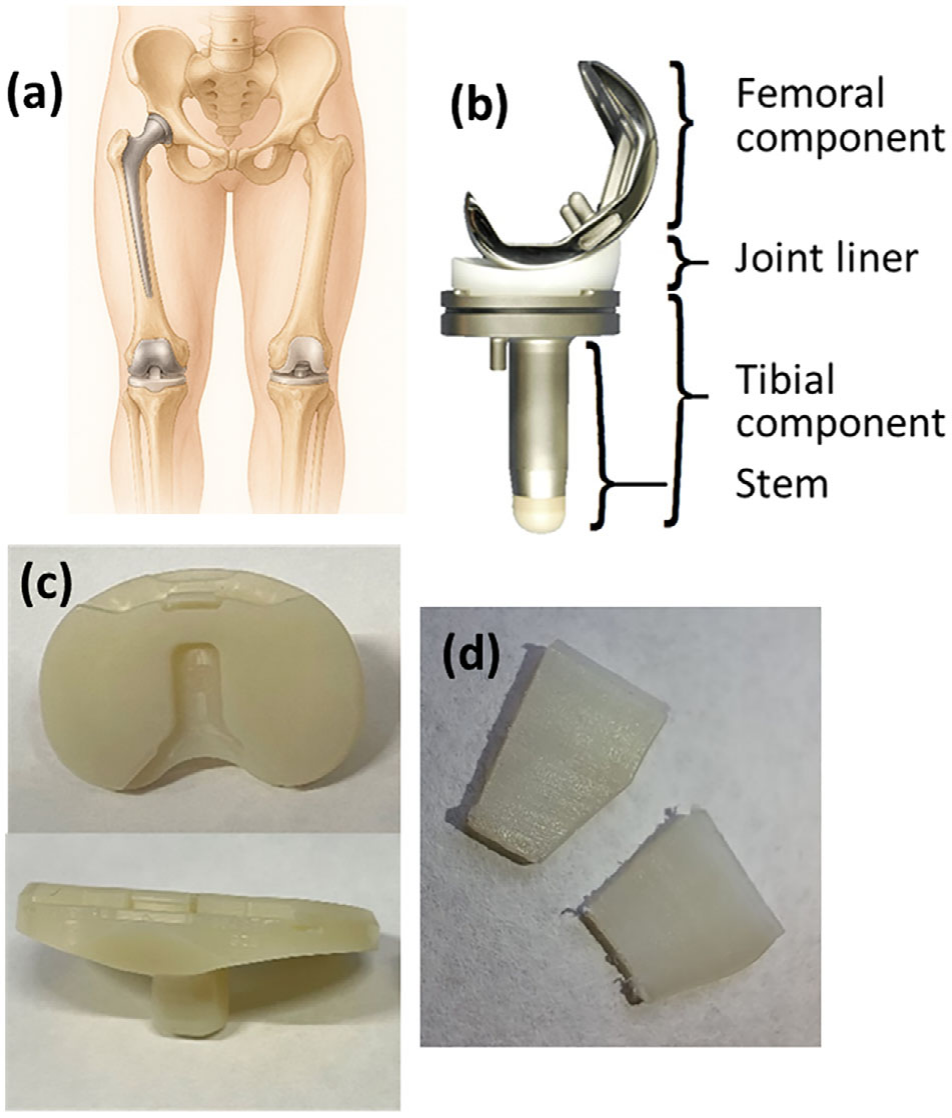
(a) Image showing knee and hip prosthesis in the body. (b) Scheme of a knee prosthesis and its different parts. (c) Pictures of a knee joint liner made out of UHMWPE. (d) Small pieces of the insert used in the impregnation experiments.

The most common type of joint prosthesis used in hip and knee surgery is the metal‐on‐plastic prosthesis due to its exceptional mechanical strength and durability, which outweighs the risks of long‐term biological reaction. Figure 1 shows a sketch of a knee and hip prosthesis in the body and details of a knee prosthesis with the different metallic and polymeric parts. The joint liner acts as an artificial cartilage and is currently made out of a highly durable plastic, such as cross‐linked Ultra High Molecular Weight Polyethylene (UHMWPE). The same material is used in the liner of the acetabular cup in hip prosthesis.

By loading the prosthesis components with antibiotics, local drug delivery at the implanted site is achieved, preventing bacteria from attaching to the implant and forming a biofilm in the early phase [8]. This provides an additional opportunity for the immune system to eliminate them, which is significantly more effective at preventing infections than systemic antibiotic administration. Furthermore, the local delivery of the drug allows for a reduction of its concentration, reducing the possible side effects of the drug in the organism, as well as contributing to fighting antimicrobial resistance [10].

Medicated implants can be prepared by loading drugs into the polymer inserts [11]. They can also be prepared by depositing, on the metal parts, an active coating containing the drug [12]. In this communication, we address the preparation of the medicated polymer parts for the slow release of the drug. Further work in the preparation of the active coating is underway.

Drug‐loaded inserts can be prepared by hot melt extrusion of the polymer parts and the drug during the prosthesis preparation [13], although this method requires high temperatures that may lead to drug degradation. They can also be prepared by solvent casting, dissolving the polymer and the drug in a solvent that is further evaporated [14], oftentimes employing toxic organic solvents that may leave solvent residues in the material. The drug can also be impregnated into the already manufactured material by soaking the material into a solution of the drug, a common procedure followed in the case of hydrogels for the preparation of hydrophilic soft contact lenses containing drugs [15]. However, in highly hydrophobic and densely cross‐linked materials such as UHMWPE, liquid‐phase impregnation presents severe limitations (need of toxic non‐polar organic solvents, poor solvent penetration, possible polymer degradation, poor compatibility with antibiotics,…), prompting the need for alternative and more effective approaches.

In view of the intrinsic limitations of liquid‐phase impregnation for UHMWPE, supercritical CO_2_ (scCO_2_) stands out as an enabling and sustainable alternative. Its successful application as a solvent for the preparation of drug‐loaded polymeric implants has already been demonstrated [8]. The appeal of scCO_2_ lies in its mild critical conditions (31°C and 73.8 bar), combined with its low cost, non‐toxicity, chemical inertness, and non‐flammability, making it a green solvent fully compliant with Principle 5 of the Green Chemistry Principles [16] by replacing toxic and volatile organic solvents. Furthermore, CO_2_ is a gas at room temperature, can be easily removed by depressurization and does not leave any residue, which contributes to preventing waste (Principle 1) and leads to safer products (Principle 4).

The Supercritical Solution Impregnation technique involves dissolving the drug in scCO_2_ and its sorption/precipitation into the support [17, 18]. In porous materials, the low interfacial tension of scCO_2_ favors wetting of narrow structures and enhances its adsorption on the pore surface [18]. In polymers, scCO_2_ diffuses through the polymer chains, carrying the drug, leading to polymer swelling/plasticization and facilitating drug sorption [8, 19, 20]. The efficiency of the supercritical fluid impregnation in polymers depends on the solubility of the drug in scCO_2_, the relative affinity of the drug to the polymer and the CO_2_ sorption capacity of the polymer [21, 22]. In both cases, the use of scCO_2_ leads to a good dispersion of the drug in the substrate.

In comparison to the impregnation using liquid solvents, scCO_2_ penetrates many polymers very well, leading to very fast dispersion and very homogeneous materials. Furthermore, the low temperatures employed avoid thermal degradation of both the drugs and the support. After depressurization, a very homogeneous distribution of the active component through the matrix is obtained, without leaving any toxic residue, and eliminating the need for drying steps [8]. The recovery or the organic solvent by evaporation and condensation is always more energy demanding than the CO_2_ cycle (compression and temperature adjustment) [23], thus the impregnation process using CO_2_ is more energy efficient than the liquid impregnation (Principle 6).

On the other hand, CO_2_ is a by‐product of industrial combustion processes that is cheap and abundant, and that can be recycled so it does not increase the net CO_2_ emissions. At the industrial level, CO_2_ and the non‐impregnated drug would be recycled, making the process economically and environmentally attractive. As an additional advantage, the antimicrobial properties of scCO_2_ [24] turn the impregnated material disinfected.

The distribution of the drug between the polymer and the CO_2_ phase is determined by the relative affinity of the drug/CO_2_, polymer/CO_2_ and polymer/drug. Depending on the polymer/drug interaction, two mechanisms have been proposed: sorption of the drug into the polymer and precipitation within the polymer matrix upon depressurization [25].

Drug‐eluting implants have been prepared successfully for ophthalmic items, sutures and scaffolds for tissue engineering, catheters and stents using this technique [8]. Furthermore, Gamse et al. [26, 27] impregnated pieces of inserts from knee and hip prostheses and an acetabular cup composed of UHMWPE with α‐tocopherol (vitamin E) to decrease their oxidative degradation. The drug was loaded into the finished product after cross‐linking and annealing. Supercritical CO_2_ impregnation was performed at 140°C–170°C and 100–300 bar. At 170°C and 300 bar, impregnation for 12 h resulted in nearly homogeneous materials containing approximately 1% α‐tocopherol [26]. Although a pilot plant for the process was reported [27], no industrial development has been identified. Similarly, a two‐step process involving doping vitamin E into UHMWPE joint implants at 120°C followed by a homogenization treatment in scCO_2_ at 100–120 bar has been proposed [28]. Homogenization treatment did not have a detrimental effect on the morphology or mechanical properties of the polymer.

Regarding the mechanical performance of the inserts after the scCO_2_ treatment, Ellis et al. demonstrated that UHMWPE could be successfully sterilized in scCO_2_ without compromising its mechanical properties [29]. Their process used scCO_2_ at 37°C and 170 bar, adding only 50 µL of H_2_O_2_ to a 40 mL reactor. Furthermore, the polymer surface showed no signs of oxidation post‐treatment, highlighting the advantages of the scCO_2_ technology.

Zhou et al. [30] have also studied the mechanical properties of as‐spun UHMWPE fibers impregnated with dyes using scCO_2_. After impregnation, the materials were drawn similarly to the non‐impregnated fibers, concluding that the supercritical impregnation process did not affect their drawability.

Building on this approach, a project is underway in our laboratory to explore the use of scCO_2_ as an impregnation and sterilization medium to treat orthopaedic prostheses to prevent infections. In this communication, we present our results on the incorporation of the antibiotic clindamycin into the polymeric parts of prostheses (inserts) using supercritical scCO_2_ as a green solvent. Clindamycin is an antibiotic proposed for infection prevention in orthopaedic prosthetic surgery, particularly in penicillin‐allergic patients [31]. We explore the effect that pressure, temperature and depressurization rate have on the drug loading and properties of the materials. The antibacterial activity of the impregnated samples was also studied.

Beyond its technical objectives, the present work addresses key Sustainable Development Goals (SDG) by contributing to Good Health and Well‐Being (SDG 3) through the prevention of post‐operative infections; to Industry, Innovation and Infrastructure (SDG 9) via the development of a sustainable processing strategy; to Responsible Consumption and Production (SDG 12) through solvent‐free manufacturing; and to Climate Action (SDG 13) by reducing solvent use and valorizing CO_2_.

## 2 Results

First, the apparent amount of clindamycin impregnated into the UHMWPE prostheses was evaluated as a function of the operating conditions. Pressure and temperature during supercritical impregnation are key parameters, since they directly affect the density of the medium. Another essential factor is the depressurization rate. Impregnation experiments were performed at 35°C–55°C and 100–200 bar for 3 h at a controlled depressurization rate ranging from 2–20 bar/min.

For the procedure to be commercially viable, apart from the antibiotic incorporation, the treatment must preserve the chemical and mechanical properties of UHMWPE. Therefore, in a second step, the effect of supercritical impregnation on UHMWPE samples was assessed using spectroscopic and thermal analyses. The purpose of the impregnation is to prevent microbial colonization of the prosthesis and thereby avoid infection once implanted. Therefore, the functionality of the impregnated samples was investigated measuring the drug release and their antimicrobial performance against a microorganism highly relevant and representative of prosthetic post‐surgical infections. Further details are provided in the Supporting Information.

### 2.1 Drug Loading in the Impregnated UHMWPE Samples and Drug Release Tests

**FIGURE 2:**
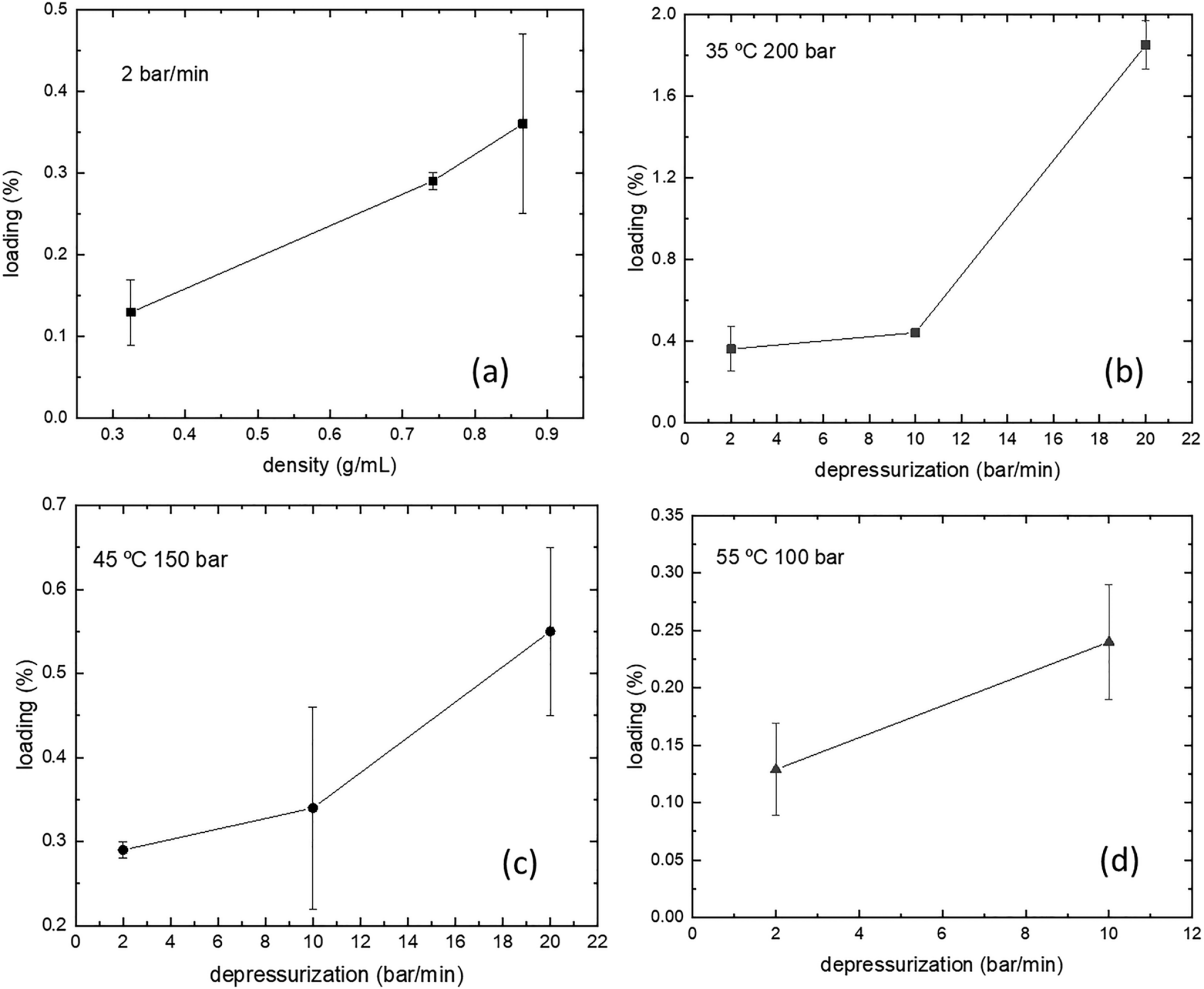
Clindamycin loading of UHMWPE prosthesis at different: (a) CO_2_ density conditions at 2 bar/min depressurization rate, (b–d) temperature and pressure conditions as a function of the depressurisation rate.

Results for the apparent drug loadings of the samples prepared at the different pressures, temperatures, and depressurization rates are given in Table 1 and plotted in Figure 2. Apparent drug loading was calculated as the percentage mass of the drug released after 24 hours with respect to the mass of the polymer inserts.

**TABLE 1:** Clindamycin apparent drug loadings into UHMWPE prosthesis pieces (%) at the different temperature, pressure and depressurization rate conditions.

T	P	CO_2_ Density [32]	Depressurization rate	Apparent drug loading
(°C)	(bar)	(g/mL)	(bar/min)	(%wt.)
35	200	0.866	2	0.36±0.11
35	200	0.866	10	0.44±0.01
35	200	0.866	20	1.85±0.12
45	150	0.742	2	0.29±0.01
45	150	0.742	10	0.34±0.12
45	150	0.742	20	0.55±0.10
55	100	0.325	2	0.13±0.04
55	100	0.325	10	0.24±0.05

Initial experiments were performed at a slow depressurization rate (2 bar/min) to avoid any damage to the prosthesis during depressurization. The experiments at the slow depressurization rate revealed low drug loadings between 0.13% and 0.44%. Further experiments were performed at different depressurization rates.

Figure 2a shows that at a fixed depressurization rate, the drug loading increases with the CO_2_ density. Although drug loadings were slightly lower than those found by Gamse et al. in the impregnation of α‐tocopherol in similar materials at much higher temperatures [26], the previous data exhibited a similar dependence on CO_2_ density.

The effect of the depressurization rate on the loading was also studied at different conditions. Figure 2b–d show that loading always increases with the depressurization rate at the different temperature and pressure conditions. Deviations in drug loading were high due to the small values and the difficulty in controlling the depressurization rate. Similar findings have been reported by Masmoudi et al. for the impregnation of intraocular lenses [33]. The highest loading of 1.85% was obtained at 35°C, 200 bar and 20 bar/min depressurization rate. These conditions correspond to the highest density and depressurization rate.

Within the experimental error, there were no significant differences in the apparent drug loadings with the thickness of the samples used (2–4 mm). However, thicker pieces may require longer impregnation times to yield homogeneous materials.

**FIGURE 3:**
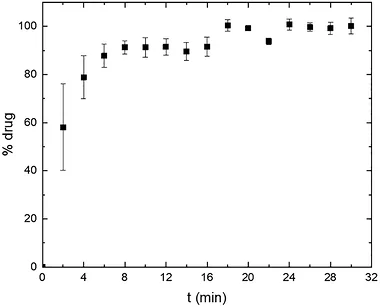
Release of clindamycin from UHMWPE prosthesis impregnated with clindamycin (0.35% apparent drug loading) in 0.01 M PBS at 37°C.

Figure 3 shows the release of clindamycin from UHMWPE prosthesis with respect to the apparent drug loading. Drug was released from the prosthesis pieces very fast into the PBS medium: ca. 90% within the first 8 min and stabilised at 18 min.

The impregnation yield, calculated as the apparent amount of drug loaded into the polymer specimens relative to the total amount of drug initially introduced into the reactor, was in the range of 5%–10% because the drug was charged in large excess. However, for scaling up the process, the drug to polymer ratio should be optimized. Also, a preliminary CO_2_ saturation stage could be added to the process, followed by a subsequent stage in which the impregnating agent was recovered from the CO_2_ by depressurisation or recirculated into the system, as it is currently done in textile dyeing [34], reducing waste generation and improving resource efficiency.

### 2.2 Impact of Supercritical Impregnation on UHMWPE Prosthesis

**FIGURE 4:**
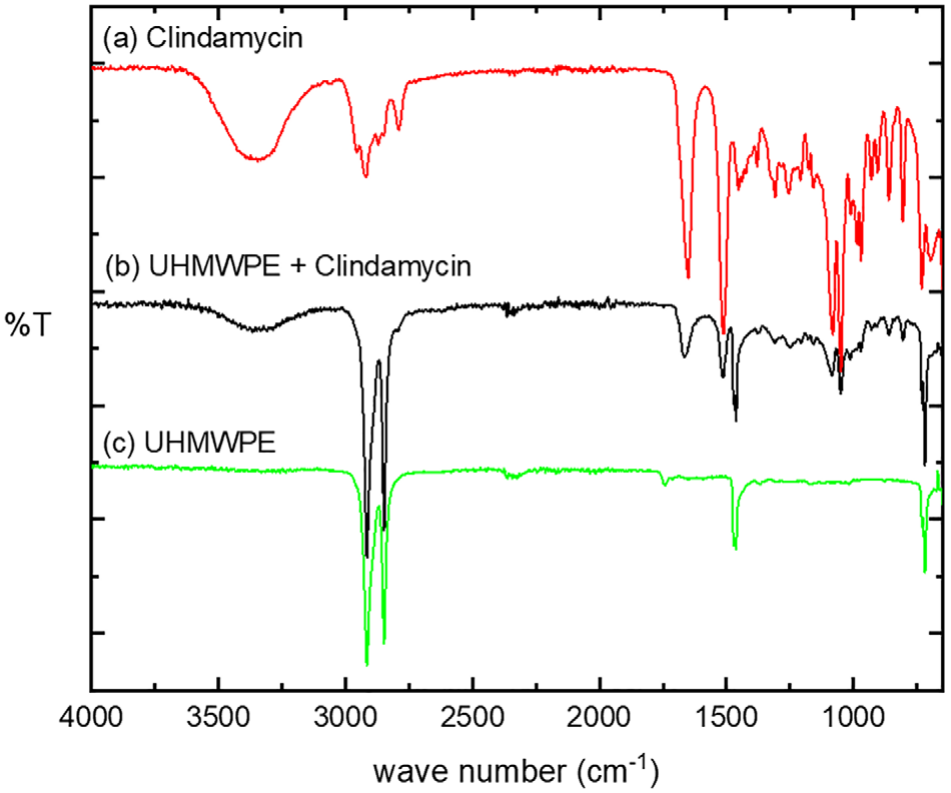
FTIR spectra of: (a) Clindamycin, (b) UHMWPE sample impregnated with clindamycin at 35°C and 200 bar depressurised at 10 bar/min and (c) UHMWPE prosthesis.

Analysis of the impregnated prosthesis was performed by FTIR spectroscopy. Figure 4 compares the spectra of a piece of the UHMWPE prosthesis, the drug clindamycin and a sample impregnated at 35°C and 200 bar, depressurised at 10 bar/min.

UHMWPE prosthesis showed the expected CH_2_ features of the symmetric and antisymmetric stretching bands at 2916 and 2848 cm^−1^, the bending bands at 1464 cm^−1^ and the rocking bands of the amorphous and crystalline regions at 718 and 730 cm^−1^, respectively. Other weak bands were observed in the starting material, suggesting the presence of additional elements in the commercial prosthesis (most likely antioxidants). On the other hand, the spectra of clindamycin base presented a broad medium stretching OH band centred at ca. 3400 cm^−1^ due to the galactose ring, CH stretching bands between 2969 and 2784 cm^−1^ and strong amide stretching and bending bands at 1650 and 1508 cm^−1^, respectively. The C‐O cyclic ether stretching band appeared at 1080 cm^−1^ and the C‐C stretching band of the pyrrolidine ring at 1048 cm^−1^. The S‐C‐H bending band at ca. 1311 cm^−1^ was not visible. The impregnated prosthesis showed clearly bands between 1750 and 650 cm^−1^ associated with clindamycin, confirming the successful impregnation of the drug into the material.

**FIGURE 5:**
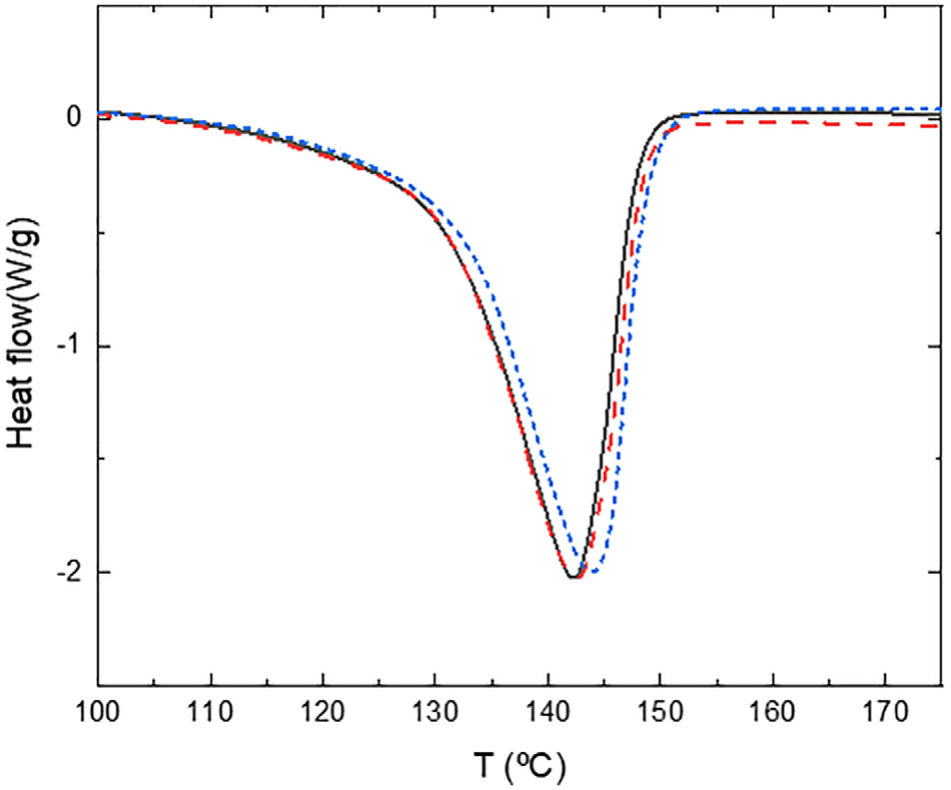
DSC thermograms of: (a) UHMWPE prosthesis (—‐) (b) UHMWPE prosthesis treated in pure CO_2_ at 35°C and 200 bar depressurised at 2 bar/min (– –) (c) UHMWPE sample impregnated with clindamycin at 35°C and 200 bar depressurised at 20 bar/min (- - -). In both cases CO_2_ treatment time was 3 h.

DSC analysis of the prosthesis before any treatment, a sample treated in pure CO_2_ at 40°C and 200 bar for 3 h and depressurised at 2 bar/min, and a sample impregnated with clindamycin at the same conditions but depressurised at 20 bar/min are shown in Figure 5. Only transitions associated to the melting of the polymer were observed.

**FIGURE 6:**
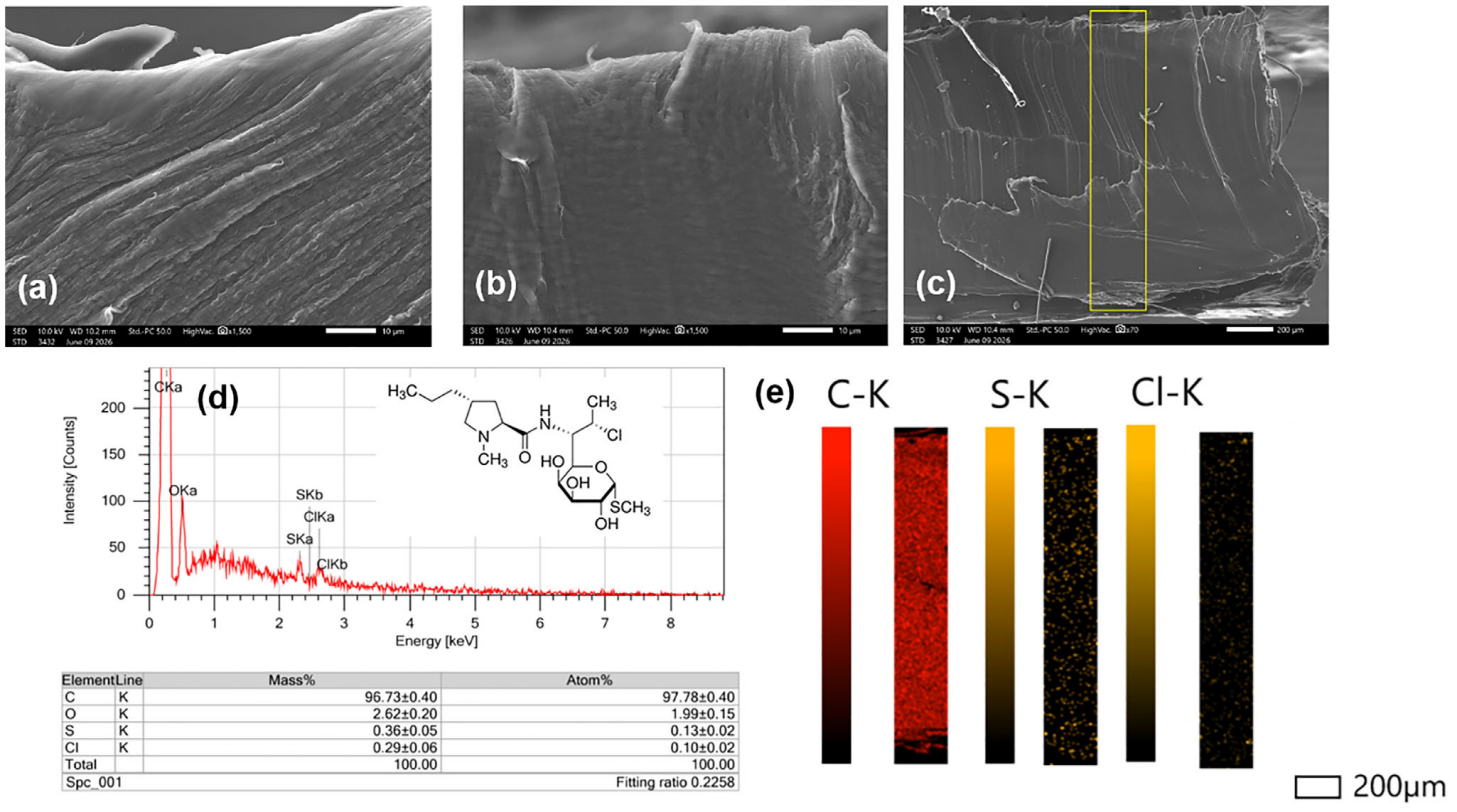
SEM images of cross‐section UHMWPE inserts (a) before impregnation, (b and c) after impregnation with CO_2_ at 35°C and 200 bar; (d) EDX analysis of the area shown in Figure b, (e) EDX elemental mapping of the rectangular section shown in Figure c. Scale bars: (a,b) 10 µm, (c) 200 µm.

Scanning electron microscopy images (SEM) of UHMWPE insert cross‐sections before and after its impregnation with clindamycin in scCO_2_ at 35°C and 200 bar are shown in Figure 6a–c. The irregular surface of the cross‐section is due to the cutting procedure. Close inspection of the borders does not show any foaming of the inserts after impregnation in CO_2_. Energy‐Dispersive X‐ray spectroscopy (EDX) analysis of the impregnated sample corresponding to Figure 6b is shown in Figure 6d. Apart from C and O, new weak peaks of S and Cl due to clindamycin are observed. These peaks do not appear in the EDX analysis of the initial UHMWPE insert. Figure 6e shows an EDX mapping of the C, O, S and Cl elements corresponding to the rectangular section highlighted in Figure 6c. S to C mole ratio was close to 1:1 in agreement with the stoichiometry of the elements in the clindamycin molecule. S and Cl appear homogeneously distributed throughout the cross section of the impregnated sample.

### 2.3 Antibacterial Properties of Clindamycin‐Loaded UHMWPE Prostheses

The antibacterial activity of the impregnated prosthesis was evaluated against *Staphylococcus epidermidis*. This bacterium was selected as the model microorganism because it is one of the leading causes of prosthesis‐ and medical device–related infections, owing to its natural colonization of human skin and its high likelihood of contaminating implants during orthopaedic surgery [35]. Its strong ability to adhere to implant surfaces and rapidly form antibiotic‐resistant biofilms makes it highly relevant for this study. Moreover, its frequent multidrug resistance justifies its use for evaluating antibiotic‐impregnated prostheses aimed at preventing postoperative bacterial proliferation.

**FIGURE 7:**
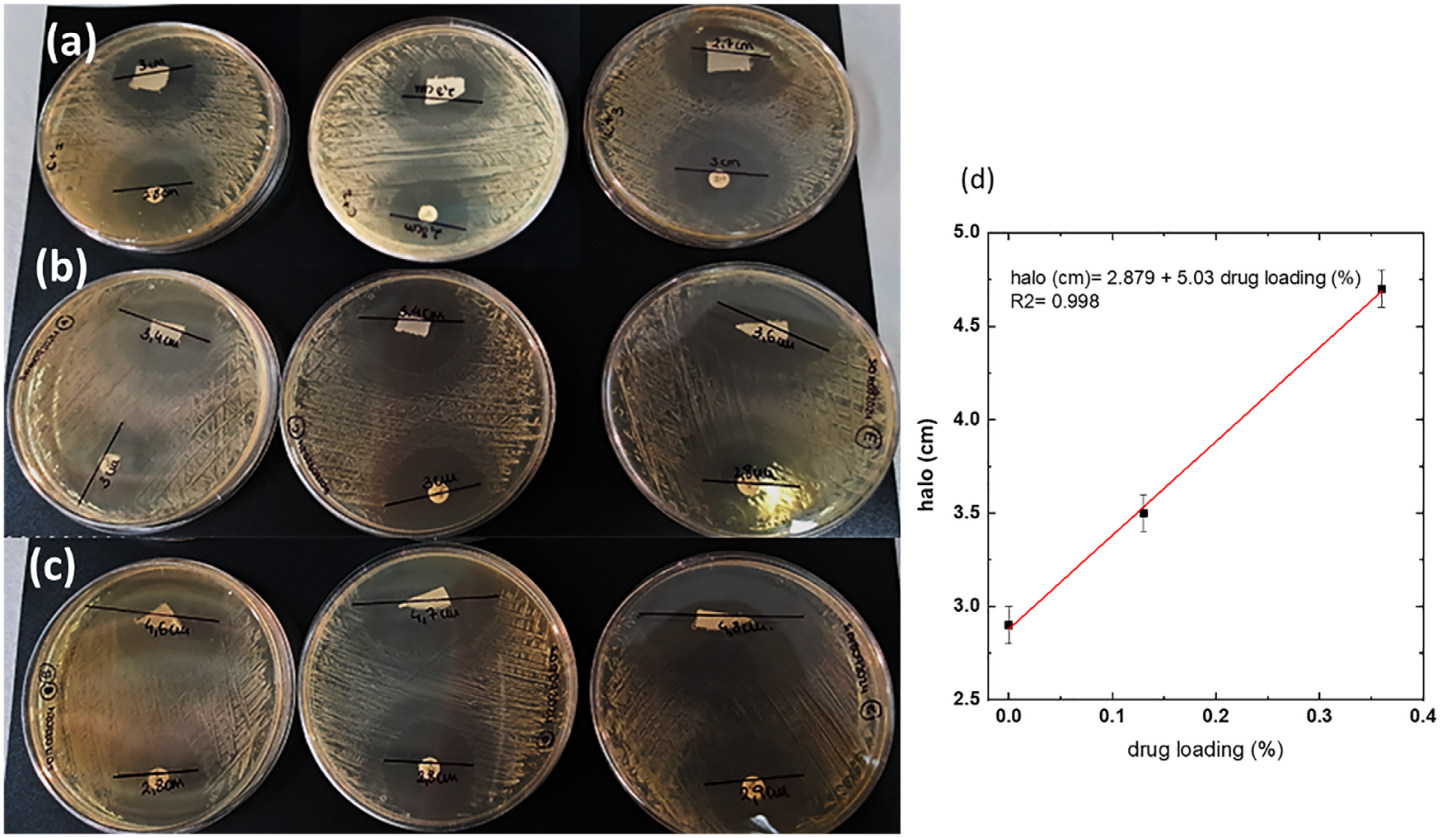
Inhibition halos formed in plates with Müller‐Hinton agar seeded with *S. epidermidis* containing pieces of: (a) Untreated prosthesis, (b) Prosthesis loaded with 0.36% clindamycin at 55°C and 100 bar, depressurised at 2 bar/min and (c) Prosthesis loaded with 0.13% clindamycin at 35°C and 200 bar, depressurised at 2 bar/min. In each plate, the halo formed around circular clindamycin standard disks is also shown (down), along with the halo formed around the irregularly shaped pieces of prosthesis (top). (d) Dependence of the halo size versus drug loading.

The antibacterial properties of the impregnated prosthesis were assessed by measuring the inhibition halos formed in plates incubated with *S. epidermidis* (Figure 7). Inhibition halos were compared to those corresponding to a clindamycin hydrochloride antibiotic disc with a loading of 2 µg.

Interestingly, pieces of the untreated prosthesis formed halos of 2.9 ± 0.1 cm, revealing the antibacterial properties of the prosthesis, possibly due to additives or antioxidants present in the material. To confirm this hypothesis, additional experiments were also performed using conventional UHMWPE plastic disks that, as expected, did not show any halo formation (see Supplementary). The halo formed in the untreated prosthesis was very similar to that formed around circular clindamycin standard disks of 2 µg (2.8 ± 0.1 cm).

Prosthesis impregnated with clindamycin at 55°C and 100 bar and 35°C and 200 bar and depressurised at 2 bar/min, with clindamycin apparent loadings equal to 0.13 (Figure 7c) and 0.36% (Figure 7b), respectively, were also studied. The halos formed were larger than those found in the untreated material, with values equal to 3.5 ± 0.1 cm and 4.7 ± 0.1 cm, increasing with the drug concentration, indicating the successful incorporation of the drug into the material. Figure 7d shows a linear dependence of the halo formed vs. the drug loading (%) in the range studied.

## 3 Discussion

The drug loading into a given support and its dependence with temperature, pressure and CO_2_ density cannot be predicted. For solutes highly soluble in CO_2_, the drug loading may decrease as the pressure and CO_2_ density increase. The solute is more soluble in CO_2_ at high pressure and density [36] and partitions preferentially to the CO_2_ phase. This is the most common situation found in the impregnation of solutes on inorganic materials when the interaction of the drug with the support is weak, and only adsorption of the solute onto the substrate is taking place [37, 38]. For polymers, however, the dependence may be different as CO_2_ sorbs into the polymers along with the solute, causing the swelling and plasticisation of the polymer. In this case, the solubility of CO_2_ into the polymer may be the most important factor. CO_2_ sorption into polymers involves the dissolution of CO_2_ into the polymer (absorption) obeying Henry's law and the adsorption on the surface obeying the Langmuir equation [39]. In both processes CO_2_ sorption always increases with pressure and CO_2_ density at moderate conditions, which could increase the drug loading. Furthermore, the specific interaction of the solute and the polymer must also be considered. Even for solutes scarcely soluble in CO_2_, Kazarian has shown that, if the polymer has functional groups that can interact with the drug by strong intermolecular forces, drug loading can be high [17].

The experiments at the slow depressurization rate revealed low apparent drug loadings certainly related to the compact nature of the cross‐linked UHMWPE material and the relatively low sorption of CO_2_ into the UHMWPE pieces [40]. Furthermore, the interaction between the drug and the PE support is weak, due to the lack of functional groups in PE.

Apparent drug loading increased with the CO_2_ density. This may be related to the relatively low solubility of the drug in CO_2_ [41] that increases always with the CO_2_ density. Furthermore, it is also due to the increase of the CO_2_ sorption into UHMWPE with pressure and density [40]. The increase of apparent drug loading with the depressurization rate at the different temperature and pressure conditions suggests a dual adsorption/precipitation mechanism. After rapid depressurization, the drug dissolved into the polymer can get trapped into the polymer network even when the interaction with the polymer is weak.

The highest apparent loading conditions corresponded to the highest density and depressurization rate. As previously discussed, the weak interaction between CO_2_ and UHMWPE (without functional groups) may provoke the drug desorption during slow depressurization, whilst fast depressurization leads to drug entrapment and an increase in the drug loading.

The impregnated prosthesis showed clearly bands associated with clindamycin, confirming the impregnation of the drug into the material. However, the melting peak of clindamycin was not observed in the DSC, indicating that the drug was dispersed in the UHMWPE pieces in an amorphous form. Furthermore, the fact that there was almost no change in the thermograms of the UHMWPE pieces treated in CO_2_ following rapid and slow depressurization indicates that there is no crystallinity change in the polymer during the impregnation procedure. The slightly higher melting temperature found in the treated sample is too small to be considered significant, corroborating that the UHMWPE material remains stable under the CO_2_ treatment [28, 29].

SEM images showed no apparent change in the polymer morphology or foaming of the polymer inserts with the impregnation process. Furthermore, EDX analysis revealed the presence of S and Cl in the inner parts of the impregnated UHMWPE insert, indicating the homogeneous dispersion of the drug into the material within the specific thickness of these samples. These results agree with previous reports by Gamse et al. [26, 27] on the supercritical impregnation of UHMWPE inserts with α‐tocopherol at 170°C and 300 bar for 12 h, which led to nearly homogeneous materials.

However, the drug release test showed only a fast discharge of the drug from the UHMWPE prosthesis pieces into the PBS medium during the first 20 min. Although the material seems to be homogeneously impregnated, because liquid solvents do not penetrate the UHMWPE insert easily, the drug release may be restricted to the most external part of the impregnated prosthesis.

Surprisingly, despite the absence of antibiotic in untreated UHMWPE, prosthesis pieces also exhibited a small inhibition halo. This phenomenon can be attributed to several factors related to the inherent properties of cross‐linked UHMWPE. First, the highly hydrophobic nature and low surface energy of UHMWPE may create an unfavorable microenvironment for bacterial adhesion and proliferation at the material‐agar interface [42]. Second, the cross‐linking process, typically performed via gamma or electron beam irradiation, generates residual free radicals and reactive oxygen species within the polymer matrix, which have been reported to exhibit mild antimicrobial properties [43]. Additionally, antioxidants such as vitamin E, commonly incorporated into medical‐grade UHMWPE to enhance oxidative stability, may contribute to this effect [44]. This was confirmed by comparing the behavior of the prosthesis pieces with that of conventional UHMWPE plastic disks, where no halo formation was observed.

Although the mass of clindamycin loaded into the prosthesis pieces was much larger than that present in the standard antibiotic disks (30‐60 times), the inhibition halos found for *Staphylococcus epidermidis* in the impregnated samples were not so different. This could be related to the slower diffusion of the antibiotic from the UHMWPE pieces in the agar, which has to diffuse from the inside of the polymer, which would limit the global mass transfer rate despite of the higher concentration driving force.

Overall, these results reinforce the potential of supercritical impregnation as a mature and scalable green technology for the development of drug‐loaded orthopaedic materials, with clear implications for infection control and sustainable medical device manufacturing. In this broader context, Spain hosts a large and active scientific and technological community working on supercritical fluid technologies, coordinated through the FLUCOMP association (https://flucomp.es/), which has played a key role in advancing sustainable processes based on supercritical CO_2_ across multiple sectors. The present contribution also aligns with the objectives of the newly established Green Chemistry section (GEQV) of the Real Sociedad Española de Química (RSEQ), highlighting the role of supercritical CO_2_ as an enabling tool for Green and Sustainable Chemistry and its translation from fundamental research to industrial practice.

## 4 Conclusions

Medicated UHMWPE inserts from orthopaedic prosthesis were successfully impregnated with the antibiotic clindamycin dissolved in supercritical CO_2_ at 35°C–55°C and 100–200 bar. The highest apparent loading (1.85%) was achieved at the highest CO_2_ density conditions, upon rapid depressurization, due to the low sorption of CO_2_ into PE and the lack of specific interaction between PE and the drug. EDX analysis showed the homogeneous dispersion of the drug into the material within the specific thickness of these samples. Drug release tests showed the fast liberation of clindamycin in the PBS medium within the first 20 min, which suggests that clindamycin is only released under these conditions from the most external parts of the prosthesis. Furthermore, the impregnated prosthesis showed antibacterial activity against *Staphylococcus epidermidis*, forming inhibition halos that increased proportionally to the drug loading. Thus, supercritical impregnation emerges as a promising strategy for the development of drug‐loaded materials in orthopaedic surgery, with clear potential to improve infection control and extend implant functionality. The technological maturity of the supercritical impregnation technique already applied at industrial level in other sectors [45] could make this process very attractive for its large‐scale implementation, paving the way toward greener medical device manufacturing.

## 5 Experimental Section

### 5.1 Materials

Clindamycin hydrochloride [C_18_H_33_ClN_2_O_5_S·HCl] (98%, Alfa Aesar) was used as the starting material to prepare clindamycin in its neutral form. Absolute ethanol (+99%, Scharlau), dimethyl sulfoxide (99%, Fisher), sodium hydroxide (98%, Panreac) and dichloromethane (99%, Honeywell) were used as received without further purification. Phosphate buffered salt solution (PBS) tablets (0.01  M phosphate) were purchased from Fisher Chemical. Deuterated water and deuterated chloroform were supplied by Acros Organics. Carbon dioxide (99.995%) was provided Carburos Metálicos.

Reagents used to prepare nutrient broths and plates for the antimicrobial activity were used as received. Meat extract, casein trypsin peptone, Müller‐Hinton broth, and agar bacteriological were obtained from Scharlau. Yeast extract and peptone were obtained from Fluka. Sodium chloride (≥ 99,5%) was purchased from Sigma‐Aldrich. The microorganism, *Staphylococcus epidermidis*, was obtained from the Spanish Type Culture Collection (CECT 231, ATCC 12228). Clindamycin hydrochloride standard antibiotic disks (2 µg per disk) were obtained from Thermo Scientific Oxoid, Fisher.

Clindamycin is commercialised in the hydrochloride and phosphate forms, but not as the neutral form due to its lower solubility in water [46]. The clindamycin neutral form, however, exhibits some solubility in sCO_2_ [41], so this was the compound used for the impregnation experiments. Clindamycin was prepared from an aqueous solution of its hydrated chloride salt (see Supplementary Material). The solubility of the neutral form of clindamycin in scCO_2_ was confirmed using a high‐pressure variable volume view, previously described [47].

Inserts from a commercial knee prosthesis made of UHMWPE, provided by Johnson & Johnson, were used as supports (Figure 1). The inserts were cut into pieces ca. 1 cm x 1 cm and various thicknesses (2–4 mm). The thinnest pieces of ca2 mm were used in the antibacterial assays.

### 5.2 Impregnation Experiments

Experiments were performed using a ca. 60 mL custom‐made high‐pressure reactor previously described [38] (see Supplementary Material). For the supercritical CO_2_ impregnation experiments, a given amount of clindamycin (between 12–40 mg) was added into the reactor along with a magnetic bar. The amount introduced corresponded to 2–3 times the reported solubility of the drug in CO_2_ at the conditions of the experiments to ensure saturation conditions [41]. Small pieces of the UHMWPE insert were placed into a basket in the upper part of the reactor to avoid direct contact with the drug (2–5 pieces). Then the reactor was heated to the selected temperature and CO_2_ was introduced from a thermostated syringe pump into the vessel up to the desired pressure, after which the system was stirred. Impregnation experiments were performed at 35°C–55°C and 100–200 bar for 3 h. After the impregnation time, the system was depressurised at a controlled rate ranging from 2–20 bar/min. Each of the conditions was assayed 3–5 times to ensure reproducibility and get a standard deviation of the measurement.

### 5.3 Quantification of Clindamycin Impregnation in Prosthesis and Drug Release Tests

The apparent amount of clindamycin impregnated into each prosthesis piece was estimated by measuring the drug released into 5 mL of dichloromethane after 24 h. Quantification was performed by UV–vis spectroscopy using a Cary 8454 spectrophotometer (Agilent Technologies). A calibration curve of the neutral form of clindamycin in dichloromethane was determined at 230 nm. Absorbance values were corrected by subtracting the baseline absorbance of the solvent at the same wavelength.

Release tests were performed on prosthesis pieces impregnated with clindamycin at 35°C and 200 bar (0.36% Clin) under sink conditions. Each piece was immersed in 20 mL of 0.01 PBS solution at 32°C under agitation (ca. 200 rpm) and samples of 2 mL were withdrawn from the solution every 2 min. Quantification was also performed by UV–vis spectroscopy, using in this case the maximum observed in PBS at 202 nm.

### 5.4 Characterization of the Impregnated Samples

FTIR spectroscopy was used to assess the successful modification of the drug to the neutral form (see Supplementary Material) and to confirm impregnation of the drug into the UHMWPE pieces. Spectra were recorded using a Perkin‐Elmer Precisely Spectrum 100 FTIR spectrometer with the universal attenuated total reflection (ATR) sampling accessory at a resolution of 4 cm^−1^.

The stability of the insert pieces after the CO_2_ treatment was studied by DSC analysis using a Q20 TA Instruments. An MT5 Mettler Toledo microbalance was used to weigh the samples, ranging between 3 and 10 mg (with an error of ± 0.001 mg). DSC were measured from −50°C to 300°C., flowing N_2_ at 50.0 mL/min. The temperature and enthalpy measurements of the calorimeter were previously calibrated using standard samples of Indium (purity > 99.999%).

A JEOL JSM IT700HR microscope was used to study cross‐sections of the UHMWPE insert pieces before and after the impregnation of the drug. The EDX of the microscope composition (60 mm^2^ DrySD detector with a resolution of 133 eV) was used for semi‐quantitative determination of sample composition. Samples for analysis were sectioned using a cutter.

### 5.5 Antibacterial Activity

To assess the resulting antibacterial activity, disk diffusion assays were conducted, comparing the effectiveness of a prosthesis impregnated with the antibiotic and a standard clindamycin disk. The disk diffusion method is widely used to evaluate antibiotic effectiveness. Guidelines for this methodology are provided by the CLSI (Clinical and Laboratory Standards Institute) and EUCAST (European Committee on Antimicrobial Susceptibility Testing) [48, 49].

The microorganism was recovered from the lyophilized state by adding nutrient broth culture medium, sown, and then kept frozen (‐80°C) until used. For reactivation, it was inoculated in an Eppendorf with 1 mL of nutrient broth for 2 h, then it was escalated in a Falcon tube to a volume of 10 mL with the same medium, where it remained for 24 h. Subsequently, 5 mL of this culture was inoculated in a bottle with 200 mL of nutrient broth and kept in a bath at a temperature of 37°C equipped with gentle lateral shaking for 24 h (VWR International, Grant Instruments, England) to achieve a concentration of bacteria necessary to obtain a suspension of *S. epidermidis* adjusted to the McFarland scale 0.5 (approximately 1.5 × 10^8^ CFU/mL).

Nutrient broth to reactivate the microorganism was prepared with 5 g of meat extract, 10 g of peptone extract, 5 g of sodium chloride and 1 L of Milli‐Q water. This preparation was heated to boiling to dissolve all components and then sterilized at 121°C for 15 min in an autoclave Med 12 (J. P. Selecta, Barcelona, Spain).

Müller‐Hinton agar in sterile Petri dishes was inoculated with the suspension of *S. epidermidis*, spreading it over the surface of the agar to ensure an even dispersion of the microorganism. Using sterile tweezers, a standard Clindamycin disk (2 µg) and a portion of the prosthesis impregnated with clindamycin were placed in different sections of the same plate. A piece of the untreated prosthesis (before impregnation) was also assayed. In addition, a commercial UHMWPE disk (2 cm diameter, 3 mm thickness) without additives (Transglass, Spain) was included as an additional negative control to assess the potential contribution of additives or antioxidants present in the prosthetic material. Plates were incubated at 37°C for 24 h under aerobic conditions. The bacterial inhibition zones around the discs and each sample were measured in mm using a Vernier calliper. Antibacterial analyses were performed in triplicate. Means and standard deviations were calculated for all data.

## Author Contributions

**Conceptualization**: A. Cabañas, L. Calvo; **Methodology**: A. Cabañas, L. Calvo, A. Salas; **Validation**: A. Salas, S. Díaz; **Formal analysis**: A. Cabañas, L. Calvo, A. Salas, S. Díaz; **Investigation**: A. Salas, S. Díaz, E. Pérez, H.K. Ruiz, R. Hafian; **Resources**: A. Cabañas, L. Calvo, P. Sánchez; **Writing – original draft**: A. Cabañas; **Writing – review & editing**: A. Cabañas, L. Calvo, E. Pérez, P. Sánchez; **Supervision**: A. Cabañas, L. Calvo; **Funding acquisition**: A. Cabañas, L. Calvo.

## Conflicts of Interest

The authors declare no conflicts of interest.

## Supporting information




**Supporting File**: gch270148‐sup‐0001‐SuppMat.docx.

## Data Availability

The data that support the findings of this study are available from the corresponding author upon reasonable request.
