# Supplementary material for: Supercritical Solution Impregnation: A Strategy to Prevent Prosthetic Infections
Source: Glob Chall. 2026 Sep 12;10(9):e70148. doi: 10.1002/gch2.70148 (PMC13570330; doi:10.1002/gch2.70148)
Supplement: Supplementary file 1 [file GCH2-10-e70148-s001.docx]

**Supercritical Solution Impregnation: A Strategy to Prevent Prosthetic Infections**

**Andrea Salas**^a^**, Sara Díaz**^a^**, Eduardo Pérez**^a^**, Helga K. Ruiz**^a^**, Rama Hafian**^b^**, Pablo Sanz**^c^**, Lourdes Calvo**^b^**, Albertina Cabañas**^a,^*

^a^Department of Physical Chemistry, Complutense University of Madrid, Spain, ^b^Department of Chemical Engineering and Materials, Complutense University of Madrid, Spain, ^c^ Hospital General Universitario Gregorio Marañón, Spain

**Corresponding author:* ***a.cabanas@quim.ucm.es***

**1. Preparation of clindamycin base**

The neutral form of clindamycin (Clin) was prepared from an aqueous solution of its hydrated chloride salt (clindamycin hydrochloride, Clin·HCl). A 0.1 M NaOH solution was added to a solution of Clin·HCl until complete precipitation. The neutral form was then extracted from the aqueous phase with dichloromethane, and the solvent was removed first by rotary evaporation and then by keeping the sample under high vacuum for a few hours. FTIR confirmed the successful modification of the hydrochloride form.

**Fig S1.** FTIR spectra of the neutral form of Clindamycin(Clin) and its chloride salt (Clin·HCl).

The DSC thermograms of the neutral form of Clindamycin and its chloride salt are compared in Fig S2. Clindamycin hydrochloride exhibits a broad endothermic peak that starts at ca. 75 ºC due to the evaporation of water trapped within the hydrated crystal lattice, followed by a melting point at 137 ºC. The drug starts decomposing at 170 ºC. These values agree with those previously reported [1]. In contrast, the neutral form of clindamycin used in the impregnation experiments is fully amorphous, showing no clear transition.

**Fig S2.** DSC of the neutral form of Clindamycin(Clin) and its chloride salt (Clin·HCl).

**2. High-pressure reactor**

Experiments were performed using a ca. 60 mL custom-made high-pressure reactor previously described [2]. The reactor shown in **Figure S3** is provided with inlet and outlet valves, a relief valve and a high-pressure gauge (all Swagelok components). The reactor was housed in a small aluminium block heated with cartridge heaters connected to a PID temperature controller (Micromega, model CN77322). J-type thermocouples were used to control the temperature of the block and measure the inner temperature of the reactor. The system was mounted onto a stirring plate. An ISCO 260D high-pressure syringe pump, thermostated at the temperature of the experiments, was used to load CO_2_ into the reactor.

**Figure S3.** Schematics of the high-pressure reactor used in the impregnation experiments showing the different parts

**3. Antibacterial activity assays on commercial UHMWPE disks.**

A commercial UHMWPE disk without additives was included as an additional negative control in the antibacterial assays to assess the potential contribution of additives or antioxidants present in the commercial prosthetic material. The absence of halo in **Figure S4**. indicates the UHMWPE material does not have antibacterial properties.

**Figure S4.** Antibacterial assays of a standard UHMWPE disk in plates with Müller-Hinton agar seeded with *S. epidermidis*.

[1]	Almoazen, H., "Development and physicochemical characterization of clindamycin resinate for taste masking in pediatrics," *Drug Development and Industrial Pharmacy* (2016).

[2]	González, J., Pérez, E., Pepczynska, M., Calvo, L., Cabañas, A., "Supercritical Solution Impregnation of naproxen into mesoporous SiO2 SBA-15," *Journal of CO2 Utilization* 73(2023), <https://doi.org/https://doi.org/10.1016/j.jcou.2023.102518>.
